# Immune Thrombocytopenic Purpura During Maintenance Phase of Acute Lymphoblastic Leukemia: A Rare Coexistence Requiring a High Degree of Suspicion, a Case Report and Review of the Literature

**DOI:** 10.4274/tjh.2014.0138

**Published:** 2015-12-03

**Authors:** Turan Bayhan, Şule Ünal, Fatma Gümrük, Mualla Çetin

**Affiliations:** 1 Hacettepe University Faculty of Medicine, Division of Pediatric Hematology, Ankara, Turkey

**Keywords:** Acute lymphoblastic leukemia, children, Immune thrombocytopenic purpura

## Abstract

Thrombocytopenia may develop in patients with acute lymphoblastic leukemia (ALL) due to myelosuppression of chemotherapy or relapse. Here we report a pediatric patient with ALL whose platelet counts decreased at the 102nd week of maintenance treatment. Thrombocytopenia was refractory to platelet infusions and bone marrow aspiration revealed remission status for ALL along with increased megakaryocytes. The cessation of chemotherapy for 2 weeks caused no increase in thrombocyte counts. The viral serology was unrevealing. A diagnosis of immune thrombocytopenic purpura (ITP) was established. After administration of intravenous immunoglobulin, the thrombocytopenia resolved. When thrombocytopenia occurs in patients with ALL in remission, ITP should be kept in mind after exclusion of the more common etiologies.

## Abstract (tr)

Akut lenfoblastik lösemi (ALL) tanılı hastalarda trombositopeni, kemoterapiye ikincil kemik iliği baskılanması veya hastalığın relapsı sonucu gelişebilir. Olgumuz ALL idame tedavisinin 102. haftasında gelişen trombositopeni nedeniyle incelendiği sırada immün trombositopenik purpura (İTP) tanısı almıştır. Trombositopeninin trombosit infüzyonuna rağmen dirençli olması üzerine yapılan kemik iliği aspirasyonunda löseminin remisyonda olduğu ve megakaryositlerin artmış olduğu görüldü. Kemoterapiye iki hafta ara verilmesine rağmen trombosit sayısında artma olmadı. Viral seroloji sonuçları aktif enfeksiyon ile uyumlu değildi. Hastaya İTP tanısı konuldu. İntravenöz immünoglobulin tedavisi ile trombositopeni düzeldi. Remisyondaki ALL hastalarında trombositopeni geliştiğinde, daha sık görülen nedenler dışlandıktan sonra İTP de akılda bulundurulmalıdır.

## INTRODUCTION

Immune thrombocytopenic purpura (ITP) is an acquired autoimmune disorder characterized by isolated thrombocytopenia due to increased platelet destruction and impaired platelet production [1]. Autoimmunity in ITP develops because of a failure in the regulatory checkpoints of the immune system, resulting in a loss of self-tolerance to platelet glycoproteins. The events that trigger this pathway are largely unknown [2]. Association of ITP with hematologic malignancies such as Hodgkin and non-Hodgkin lymphoma or chronic lymphocytic lymphoma is a well-known phenomenon. ITP has also been reported to accompany acute lymphoblastic leukemia (ALL), albeit extremely rarely [3]. Herein we report a patient with ALL who developed ITP during maintenance therapy for ALL.

## CASE PRESENTATION

A 3-year-old girl was admitted with fever, bone and joint pain, and malaise. Complete blood count showed a hemoglobin level of 7.4 g/dL, platelet count of 97x109/L, and white blood cell count of 3.8x109/L with 34% blasts on the peripheral blood smear. Bone marrow aspiration revealed CALLA (+) pre-B cell ALL. A modified St. Jude Total XV protocol was initiated with institutional modifications in the induction phase concerning the dose of steroids, and remission was achieved [4]. Maintenance treatment was planned according to the patient’s low risk status [4]. Nothing was remarkable up to the 102nd week of maintenance. After the 68th week of treatment, maintenance included weekly parenteral methotrexate (40 mg/m2) and daily oral 6-mercaptopurine (75 mg/m2/day) with pulses of dexamethasone and vincristine every 4 weeks until the 100th week, after which only 6-mercaptopurine and methotrexate were given. At that time, routine blood count showed hemoglobin of 12.8 g/dL, white blood cell count of 5.4x109/L, and platelet count of 43x109/L. Physical examination revealed no hepatosplenomegaly. She was free of bleeding symptoms despite ecchymoses of the lower extremities. Treatment was ceased for 2 weeks and, at the end of 2 weeks of follow-up, thrombocytopenia persisted. Since the platelet count had decreased to 16x109/L, irradiated and filtered platelet transfusion was administered, but the next day the platelet count was found to still be as low as 21x109/L. Viral tests for parvovirus B19 polymerase chain reaction (PCR), Epstein-Barr virus PCR, and cytomegalovirus PCR were all negative. Antinuclear, antidouble-stranded DNA antibodies and direct Coombs test were negative. Vitamin B12 and folate levels were within normal ranges. In order to exclude the possibility of associated hemophagocytic lymphohistiocytosis, testing of plasma fibrinogen, serum triglyceride, and ferritin levels was ordered and all were found to be within the normal range. Bone marrow aspiration was performed in order to exclude relapse of ALL. The bone marrow examination revealed a cellular bone marrow in remission for ALL with erythroid hyperactivity and increased megakaryocytes (up to 9-10/field at 10x magnification). A diagnosis of acute ITP was established and intravenous immunoglobulin (IVIG) therapy was given (1 g/kg/day, for 1 day). Three days after IVIG treatment, platelet count was found to have increased to 272x109/L. During follow-up, thrombocytopenia showed no recurrence, despite continuation of the maintenance treatment without any modification. Informed consent was obtained.

## DISCUSSION AND REVIEW OF THE LITERATURE

Thrombocytopenia seen in patients with ALL is generally secondary to chemotherapy or relapse of primary disease. Both of these conditions manifest with reduced platelet production [1]. Impaired megakaryocytopoiesis may also be seen in ITP, but commonly accelerated destruction of platelets results in increased megakaryocytes in bone marrow as a distinctive finding of ITP [1,5]. In our patient, we did not check for antiplatelet antibodies; however, bone marrow findings, as well as the response of thrombocytopenia to IVIG treatment, were strongly suggestive for the diagnosis of ITP.

Classically, the pathophysiology of ITP is attributed to opsonization of platelets by immunoglobulin G antibodies and then phagocytosis and destruction by macrophages in the reticuloendothelial system within the spleen [5]. T cell-mediated immunity is also important in ITP pathogenesis [2]. Regulatory T cells (Treg cells) marked by CD4+CD25+Foxp3+ have essential roles in self-tolerance by suppression of humoral and cellular immunity response [6]. Treg cells have been blamed for a role in ITP. Reduction in number and/or function of circulating Treg cells in ITP patients has been shown in several reports [1,5]. Increased numbers of CD4+ Th17 cells and higher levels of T cell-related cytokines are other T cell abnormalities detected in ITP [5].

In the English-language literature, 9 pediatric patients who developed ITP subsequent to a diagnosis of ALL were reported in 7 reports; 6 of them were on chemotherapy and 3 patients’ ITP developed after cessation of chemotherapy (Table 1) [3,7,8,9,10,11,12]. It seems paradoxical to diagnose ITP in patients with ALL who are under extensive immune suppression with chemotherapeutics for the primary disease. Because of the intensive chemotherapy used in ALL, autoimmune diseases have rarely been reported among patients with ALL who are under treatment [13]. Of the reported cases, ITP was detected during the maintenance period in 4 of the patients, in 1 patient after reinduction, in 1 patient after induction therapy, and in 3 patients after cessation of chemotherapy [3,7,8,9,10,11,12]. In the majority of these reports, ITP was diagnosed during treatment with 6-mercaptopurine, similar to our case [3,8,9,10]. In 2 of these reports, 6-mercaptopurine treatment was continued without recurrence of ITP; in 1 case, due to resistant thrombocytopenia, maintenance therapy was administered with the support of IVIG; and in 1 report, continuation of 6-mercaptopurine after development of ITP was not stated clearly [3,8,9,10]. 6-Mercaptopurine is a purine nucleoside analogue that disturbs DNA synthesis and induces apoptosis [14]. Purine nucleoside analogues cause profound depletion of T cells [15]. Consequently, CD4+CD25+Foxp3+ cell counts also decrease, and this will result in immune dysregulation. This cascade has been thought of as a mechanism of ITP seen in ALL [9,10]. In the literature, 2 patients were reported to have developed ITP after treatment with cyclophosphamide [9,12]. Cyclophosphamide also has suppressive effects on Treg cells, similar to purine analogues, and this may support the association of Treg cells with ITP in patients with ALL [9].

In conclusion, newly developed persistent thrombocytopenia in patients with ALL may indicate ITP. After exclusion of other common causes including recurrence of the primary disease, chemotherapy-related myelosuppression, folate deficiency, or viral etiologies, the coexistence of ITP should be kept in mind as a rare etiology for unexplained thrombocytopenia in order to initiate appropriate treatment as early as possible.

## Figures and Tables

**Table 1:**
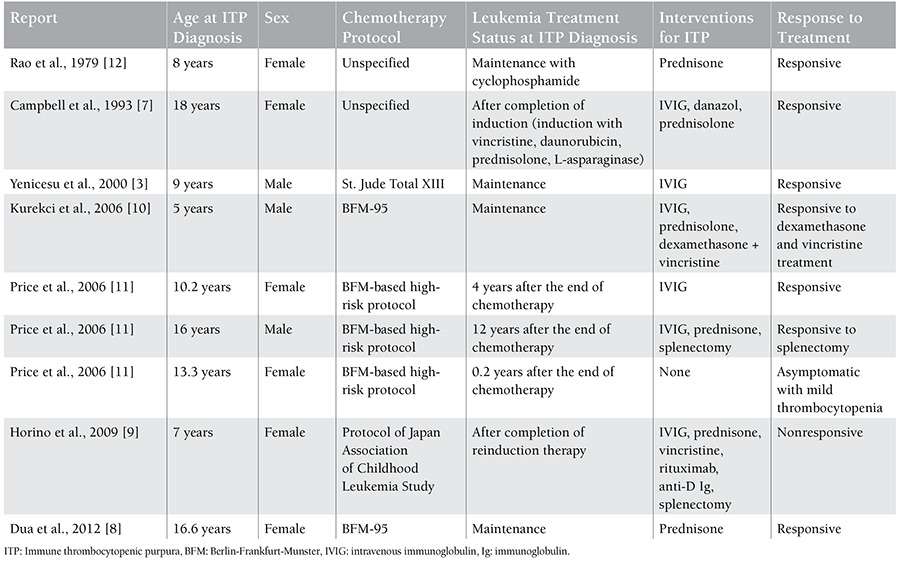
Reported pediatric cases with immune thrombocytopenic purpura subsequent to a diagnosis of acute lymphoblastic leukemia.
